# Methotrexate-Associated Nonalcoholic Fatty Liver Disease with Transaminitis in Rheumatoid Arthritis

**DOI:** 10.1155/2014/823763

**Published:** 2014-05-28

**Authors:** Rajalingham Sakthiswary, Grace Yin Lai Chan, Ee Tzun Koh, Khai Pang Leong, Bernard Yu Hor Thong

**Affiliations:** ^1^Department of Rheumatology, Allergy and Immunology, Tan Tock Seng Hospital, Singapore 308433; ^2^Department of Medicine, Universiti Kebangsaan Malaysia Medical Centre, Jalan Yaacob Latif, Bandar Tun Razak, 56000 Cheras, Kuala Lumpur, Malaysia

## Abstract

*Background*. The aim of this study was to determine the risk factors of MTX-associated nonalcoholic fatty liver disease (NAFLD) with transaminitis in a cohort of rheumatoid arthritis (RA) patients from Singapore. *Methods*. Patients who developed ultrasound proven NAFLD with transaminitis while on MTX therapy were identified. The demographic and clinical characteristics of the above patients (cases) were compiled and compared with age- and gender-matched controls who were RA patients on long standing MTX therapy without any episode of transaminitis. *Results*. Among the 978 patients who had received MTX, the prevalence of MTX-associated NAFLD was 4.7% (46 patients). Compared to the controls, the cases had significantly higher mean cumulative dose of MTX (4.03 ± 2.25 g versus 10.04 ± 9.94 g, *P* ≤ 0.05), weekly dose of MTX (11.3 ± 4.8 mg versus 13.1 ± 4.4 mg weekly, *P* = 0.033), and fasting blood glucose (*P* = 0.029). Following multivariate regression analysis, only cumulative dose of MTX remained significant (*P* = 0.015). Among the cases, the cumulative dose of MTX was found to have a significant positive correlation with the alanine transaminase (ALT) level (*P* < 0.05, standardised beta coefficient 0.512). *Conclusion*. The cumulative dose of MTX was the only independent predictor of MTX-associated NAFLD with transaminitis.

## 1. Introduction

Methotrexate (MTX) is recommended as the first-line disease-modifying antirheumatic drug (DMARD) by the European League Against Rheumatism (EULAR) and the American College of Rheumatology (ACR) [1, 2] in the treatment of rheumatoid arthritis (RA). Patients with RA tend to stay on MTX for years, making its long-term safety profile a major concern. A systematic review by Salliot and van der Heijde [3] identified elevation of liver enzymes as the second most common adverse event with MTX therapy (20.2%) following gastrointestinal side effects. The spectrum of liver involvement with MTX therapy is rather broad and ranges from mild transaminitis [4, 5] to liver failure [6, 7].

Nonalcoholic fatty liver disease (NAFLD), which refers to demonstration of hepatic steatosis by imaging or biopsy in the absence of heavy alcohol consumption [8], has been associated with MTX therapy [9, 10]. It is believed to be related to folate antagonism in the hepatic tissues which have a high cellular turnover and, hence, a high requirement of purines, thymidine, and methionine [11]. Liver biopsy specimens from RA patients have demonstrated hepatic folate deficiency and accumulation of MTX-polyglutamates [12]. The other culprit mechanisms involved are probably inhibition of purine metabolism, polyamine synthesis, and homocysteine metabolism [11].

To date, there are no reliable tests that predict the development of NAFLD with MTX therapy. The preliminary results from a few studies on genetic polymorphisms in the metabolic pathways of MTX have proposed the influence of pharmacogenetics in NAFLD on RA. Urano et al., for instance, reported that C677T polymorphism increased the likelihood of MTX toxicity [13, 14]. Unfortunately, such genetic analysis may not be feasible in all parts of the world. Till today, there is still profound lack of data on clinical determinants and predictors of NAFLD among RA patients on MTX. As such, this study is an attempt to fill the knowledge gaps in this context.

## 2. Methodology

### 2.1. Patients

The RA registry of Tan Tock Seng Hospital consists of 1105 patients who were recruited consecutively from year 2006 to year 2013. This registry contains demographic and clinical details of all RA patients followed up at our centre including their comorbidities and disease activity scores. The study visits were at 3 monthly intervals. We performed a retrospective review of the medical records of patients who had ever received MTX therapy for a minimum period of 3 months. The exclusion criteria were patients who abuse alcohol; presence of chronic hepatitis B, chronic hepatitis C, autoimmune hepatitis, primary biliary cirrhosis, and all other forms of chronic liver disease.

Details regarding the demographic characteristics, treatment dosing, and laboratory follow-up were available from a single computer database. Liver enzymes were recorded every study visit as part of the routine care. Patients who developed ultrasound proven NAFLD with transaminitis (any degree of raised alanine transminase (ALT) and/or aspartate transaminase (AST)) while on MTX therapy were identified. The demographic and clinical characteristics of the above patients (cases) were compiled and compared with age- and gender-matched controls who were RA patients on long standing MTX therapy without any episode of transaminitis. Patients with NAFLD or transaminitis alone were excluded. The ratio of cases to controls was fixed at 1 : 2. For the cases, the time point used as reference for the recording of data was during the peak of ALT and/or AST whereas for the controls, we used the data from the last study visit.

NAFLD was diagnosed based on characteristic ultrasonographic features which include diffuse hyperechogenicity of the liver in relation to the kidneys and ultrasound beam attenuation and absence of significant alcohol consumption. Liver biopsy for histological evidence of hepatic steatosis was not required.

### 2.2. Assessment for Nonalcoholic Fatty Liver Disease

According to the American Association for the Study of Liver Diseases guidelines in 2012, the diagnosis of NAFLD requires evidence of hepatic steatosis, either by imaging or by histology in the absence of significant alcohol consumption. Although an abnormal liver function test is not a mandatory criterion, for the purpose of this study we only included patients with any degree of transaminitis and NAFLD. From our observation, transaminitis was the main prompt for ordering hepatic ultrasonography among our rheumatologists. In all the cases, hepatic steatosis was diagnosed on the basis of characteristic sonographic features which include diffuse hyperechogenicity of the liver in relation to the kidneys and ultrasound beam attenuation [15]. Ultrasonography has good sensitivity and specificity of up to 90–95% for detecting moderate to severe hepatic steatosis [15]. Details on semiquantitative sonographic scoring for the degree of hepatic steatosis, that is, mild, moderate, or severe, were not available but this form of grading is sonographer-dependent and therefore remains subjective.

### 2.3. Statistical Analysis

All statistical analyses were conducted using the Statistical Package for Social Sciences (SPSS) package for Windows version 21. Continuous variables were expressed as mean ± SD. Differences between the two groups (the cases and the controls) were analysed using the chi square test and Fisher's exact test for categorical data, and the Mann-Whitney test and independent *t*-test were used for continuous variables. The odds ratio (OR) was determined using multivariate logistic regression for variables with significant *P* values (*P* < 0.05) on univariate analysis. The cutoff value of the cumulative dose of MTX for the development of NAFLD with transaminitis was extrapolated by calculating the area under the receiver operator characteristic curve (AUC).

## 3. Results

### 3.1. Prevalence of NAFLD with Transaminitis and Baseline Characteristics of the Cases and Controls

Out of 1105 patients, only 978 patients had ever received MTX therapy. A total of 112 patients (11.4%) had developed transaminitis while on MTX therapy. The prevalence of MTX-associated NAFLD with transaminitis was 4.7% (46 patients). The demographic and clinical characteristics of the cases and controls are summarised in Table 1. Most of the patients were females (80.1%). There were no significant differences between the two groups in terms of demographic, disease activity, median dose of concomitant disease-modifying antirheumatic drugs (sulfasalazine, prednisolone, leflunomide, and hydroxychloroquine), lipid profile, and renal function. However, compared to controls, cases had significantly higher cumulative dose of MTX (*P* < 0.05), mean weekly dose of MTX (*P* = 0.033), and fasting blood sugar (FBS) (*P* = 0.029).

### 3.2. Multivariate and Correlation Analysis

On multivariate logistic regression of these significant variables, the cumulative dose of MTX was the only predictor of NAFLD with transaminitis (*P* = 0.015).  Table 2 outlines the multivariate analysis findings of significant variables on univariate analysis, that is, cumulative dose of MTX and mean dose of MTX and FBS. Among the cases, the cumulative dose of MTX was found to have a significant positive correlation with the ALT level (*P* < 0.05, standardized beta coefficient 0.512).

### 3.3. Cutoff Value for the Cumulative Dose of Methotrexate in Predicting NAFLD with Transaminitis

We determined the cutoff value for the cumulative dose of MTX in predicting NAFLD with transaminitis using the ROC curve analysis. We found that 5503.75 mg of the cumulative dose of MTX was a predictor in this regard with the following statistical values: AUC 0.684, 95% confidence interval 0.777 to 0.792, *P* = 0.001, sensitivity 54.5%, specificity 78.3%.

## 4. Discussion

To the best of our knowledge, this is the first study describing the predictors of MTX-associated NAFLD with transaminitis in RA. In this large cohort of patients, the prevalence of the above condition was 4.7%. This figure is somewhat lower than expected as the reported prevalence of NAFLD in large population-based studies ranges from 11% to 24% [16, 17]. Methodological, ethnic, and genetic variations may account for the discrepancy between our finding and the aforementioned studies.

In an epidemiological study by Foster et al. [16], for instance, NAFLD was defined as liver spleen ratio < 1 from CT measurements. Although Niaz et al. [17] used a similar study design to ours, the study population comprised only young male individuals. The vast majority of our subjects, on the other hand, were females. Unfortunately, there is no data on the incidence of MTX-associated NAFLD with transaminitis in RA to make a strict comparison here. However, a systematic review by Visser and van der Heijde [18] pointed out that the incidence of transaminitis in the first three years of MTX use was 13/100 patient-years with a cumulative incidence of 31%.

This study has identified cumulative MTX dose as an independent predictor of MTX-associated NAFLD with transaminitis. Besides, this variable had a significant dose-response relationship with the severity of the transaminitis based on the ALT levels. Walker et al. [19] and Visser and van der Heijde [18] reported that liver toxicity, cirrhosis, and failure were MTX-dose related in RA, which were parallel to our finding. The exact mechanism which links cumulative MTX dose to NAFLD remains unclear. Several processes and pathways have been implicated in MTX related hepatotoxicity but folate antagonism probably takes the lead role [11, 12]. This theory, however, lacks conclusive proof. Cáliz et al. demonstrated that the C677T polymorphism in the MTHFR gene is associated with MTX toxicity in a Spanish RA population [20]. Single nucleotide polymorphisms (SNP) in the genes encoding enzymes in the MTX cellular pathway have been implicated in determining both MTX efficacy and toxicity [21]. In a recently published meta-analysis, however, the authors concluded that neither C677T nor A1298C gene polymorphism showed association with MTX toxicity [22]. From a research standpoint, the finding of a significant relationship between cumulative MTX dose and MTX-associated NAFLD does not imply causation of the latter by the former.

Based on this study, no other demographic or clinical variables were found to be a determinant of MTX-associated NAFLD with transaminitis. Although the mean fasting blood sugar was higher among the cases on univariate analysis, it was within nondiabetic range (5.88 ± 1.94 mmol/L). The number of diabetics did not differ significantly between the cases and controls although diabetes mellitus is a well recognised risk factor for NAFLD [23, 24]. Besides, several studies have suggested that leflunomide in combination with MTX may result in up to 4-fold elevation of liver enzymes [25, 26]. However, in this study, the concomitant use of other DMARDs, namely, leflunomide, sulfasalazine, and hydroxychloroquine, did not appear to influence the development of NAFLD with transaminitis. The difference in the prednisolone dose was almost significant (*P* = 0.052) at univariate analysis. The subjects with MTX-associated NAFLD with transaminitis had lower daily dose of prednisolone. The lower steroid dose requirement could be partially explained by the higher dose of MTX in this group. Although the duration of MTX therapy and the average weekly MTX dose were not significant factors on their own, it is noteworthy that the cumulative MTX dose was determined by these variables. Hence, the importance of both of these parameters cannot be understated.

This study has several limitations. Firstly, the diagnosis of NAFLD is based on sonographic findings and lacks histological confirmation. Although ultrasonography has good sensitivity and specificity in the detection of moderate and severe hepatic steatosis (90–95%), the performance is markedly reduced when the hepatic fat infiltration is less than 33% on liver biopsy [15]. This means that our study could have underestimated the actual prevalence of NAFLD. Secondly, baseline ultrasonography prior to the commencement of MTX was not available. Despite its noninvasiveness, ultrasound of the liver is not a routine investigation in the care of our RA patients who have no underlying suspected liver disease.

In conclusion, the prevalence of MTX-associated NAFLD with transaminitis in this series is rather low. The cumulative dose of MTX was the only independent predictor and it correlated closely with the ALT levels which were reflective of the severity of the transaminitis. We acknowledge that the results from our study may not be generalisable to other populations owing to the genetic influences on MTX metabolism. Further longitudinal studies would hopefully validate our preliminary findings.

## Figures and Tables

**Table 1:** Comparison of characteristics between cases and controls.

Demographic variables	Cases (*n* = 46)	Controls (*n* = 92)	*P* value
Age	54.28 ± 9.50	60.45 ± 10.65	0.487
Ethnic groups			
Chinese	36 (78.3%)	75 (81.5%)	0.891
Malays	5 (10.9%)	9 (9.8%)	
Indians	5 (10.9%)	8 (8.7%)	
Gender			
Female	37 (80.4%)	72 (78.3%)	0.768
Male	9 (19.6%)	20 (21.7%)	
Seropositive (rheumatoid factor positive)	25 (54.3%)	51 (55.4%)	0.184
Body mass index (kg/m^2^)	25.82 ± 6.16	24.50 ± 4.33	0.312
Disease duration (months)	116.76 ± 87.86	108.60 ± 20.78	0.402
Duration on MTX (months)	50.60 ± 23.63	47.31 ± 26.93	0.531
Disease activity score (DAS 28)	2.03 ± 0.83	2.28 ± 0.73	0.078
Diabetes mellitus	13 (28.3%)	31 (33.7%)	0.566
Methotrexate dose (mg/week)	13.09 ± 4.35	11.27 ± 4.75	**0.033**
Cumulative methotrexate dose (mg)	10,037.09 ± 9,939.05	4,026.80 ± 2,251.83	**<0.050**
Patients treated with prednisolone	19 (41.3%)	35 (38.0%)	0.716
Prednisolone dose (mg/day)	1.33 ± 1.55	2.20 ± 2.79	0.052
Patients treated with sulfasalazine	18 (39.1%)	27 (29.3%)	0.255
Sulfasalazine dose (g/day)	2.00 (2.0)	2.00 (2.5)	0.143
Patients treated with hydroxychloroquine	16 (34.8%)	35 (38.0%)	0.711
Hydroxychloroquine dose (mg/day)	200.00 (400.0)	300.00 (400.0)	0.251
Patients treated with leflunomide.	11 (23.9%)	25 (27.2%)	0.837
Leflunomide dose (mg/day)	20.00 (10.0)	20.00 (10.0)	0.887
On statin therapy	12 (26.1%)	16 (17.4%)	0.231
LDL (low density lipoprotein) (mmol/L)	3.12 ± 0.72	3.08 ± 0.844	0.851
HDL (high density lipoprotein) (mmol/L)	1.44 ± 0.40	1.52 ± 0.44	0.425
Triglycerides (mmol/L)	1.22 ± 0.65	1.17 ± 0.58	0.680
Total cholesterol (mmol/L)	5.12 ± 0.81	5.14 ± 0.88	0.917
Fasting blood sugar (mmol/L)	5.88 ± 1.94	5.14 ± 0.64	**0.029**
Creatinine (*μ*mol/L)	69.67 ± 28.51	66.41 ± 19.70	0.434
Glomerular filtration rate (mls/minute)	86.20 ± 26.89	87.80 ± 29.13	0.755

Values are expressed as number (%), median (range), or mean ± SD.

**Table 2:** Multivariate analysis of independent prediction of NAFLD with transaminitis.

Variable	Odds ratio	*P* value	95% confidence interval	
			Lower	Upper
Cumulative methotrexate	1.035	**0.015**	0.092	1.051
Methotrexate dose	0.944	0.337	0.840	1.062
Fasting blood Sugar	0.564	0.129	0.270	1.180
